# Factors sustaining legitimacy of smoking in Vocational Education and Training (VET) schools: a qualitative needs assessment

**DOI:** 10.1186/s12889-024-18033-8

**Published:** 2024-03-04

**Authors:** Dina Danielsen, Johan Lerbech Vinther, Ditte Heering Holt, Gitte Sofie Jakobsen, Lotus Sofie Bast, Susan Andersen

**Affiliations:** 1 National Institute of Public Health, University of Southern Denmark, Copenhagen, Denmark; 2 Department of Public Health, University of Copenhagen, Copenhagen, Denmark

**Keywords:** Smoking prevention, Smoking reduction, Low SES youth, VET schools, Needs assessment, Legitimacy of smoking

## Abstract

**Background:**

Most adult smokers started smoking in their teenage years, which increases the risk of nicotine dependence. In Denmark, there is a high prevalence of youth smoking among students in Vocational Education and Training (VET). However, reducing and preventing smoking in this group is a major challenge. This article presents a needs assessment aimed to explore factors sustaining legitimacy of smoking in VET schools and consider the measures needed to prepare VET schools’ implementation of smoking reduction and prevention interventions.

**Methods:**

Participant observations were conducted in four VET classes representing three VET schools in Denmark with a duration of four days each. Individual, semi-structured interviews were conducted with teachers, managers, and a student advisor, as well as four focus groups with a total of 20 students. Interviews were audio-taped, transcribed, and the data material was analyzed following Malterud’s systematic text condensation.

**Findings:**

Factors that helped sustain legitimacy of smoking in VET schools included a positive and normalized attitude towards smoking at home and among friends, an understanding of smoking as an integral and expected practice in VET professions and schools, a perceived reliance on smoking as an icebreaker in new social relations and as a pedagogical tool, and smoking as a habit and a means to deal with boredom and stress relief.

**Conclusions:**

The factors sustaining legitimacy of smoking in VET schools are reciprocal and call for smoking reduction and prevention intervention efforts which consider and address social influence, habitual behavior, and psychological needs, as well as changes at the policy level.

## Background

During the last two decades, overall, the prevalence of tobacco smoking in Western countries has decreased in all age groups. The decline among youth in Denmark stagnated for a period around 2013 until 2017. However, the latest national data showed that smoking among 16–24-year-olds is declining again [1]. Nonetheless, tobacco is still one of the single most important preventable determinants for death and diseases worldwide [2]. In Denmark, smoking is a contributing factor to approximately 13,600 deaths annually, which corresponds to one in four deaths [3].

Evidence shows that most people who smoke start in youth [4], and those initiating smoking at a young age are at higher risk of developing nicotine dependence and less likely to quit [5]. Accordingly, researchers have highlighted the importance of focusing on youth as a period of increased vulnerability to experimentation and escalation of smoking [6]. This emphasizes the need for prevention of escalation in dependence to prevention in early adolescence. Although smoking is initiated in early adolescence, stable patterns of smoking are often not fully established in adolescence and young adulthood [6]. In this respect, youth is an important period in life for disrupting the pathway of escalating health-behaviors, including smoking practices.

The latest report on tobacco use from the World Health Organization (WHO) confirms the existence of global social inequality in smoking initiation and prevalence [7]. In Denmark, a similar trend is observed, where heavy daily smoking is more prevalent among low SES youth [1, 8, 9]. Socioeconomic differences in smoking among Danish youth is illustrated by differences in smoking prevalence among high school students, primarily with a high SES, and Vocational Education and Training (VET) students, primarily with a low SES background [10, 11]. National representative surveys from 2019 showed that 29% of VET students smoked daily whereas the proportion was 9% among Danish high school students [10–12]*.* Part of this difference may be ascribed to a higher proportion of youth with lower socioeconomic status attending VET schools compared with high schools [13]. This underscores the significance of VET schools as an important setting for interventions aimed to reducing inequality in smoking. Moreover, evidence suggests that smoking cessation is more difficult among individuals from low SES groups, which further increases existing inequalities [14, 15]. A systematic review identified multifaceted barriers to smoking cessation within vulnerable groups, including that smoking was related to stress management, lack of support from health- and other service providers, as well as high prevalence and acceptability of smoking in the community [16]. This highlights the complex interplay of factors contributing to the persistence of smoking habits, particularly among populations facing heightened vulnerability. A previous intervention study in Danish VET schools found that smoking was considered an acceptable practice among students and teachers, thus suggesting that existing institutional practices likely reproduced or even strengthened the formation of smoking communities [17]. The study also identified a lack of priority among VETs to support health promoting student communities, partly due to school managers that were preoccupied in competing tasks and demands, such as planning a comprehensive future educational reform on the area. Moreover, few social and physical environments were in place to inspire to non-smoking activities or help students to establish new relations and friendships. In this respect, smoking was a central way to interact and create social relations among students [17, 18]. In Denmark, the VET system offers more than 100 different educational paths directed towards skilled professions [19]. The prescribed duration is about three to four years, and the system offers degrees within four main subject areas, namely 1) Care, Health, and Pedagogy (CHP), 2) Administration, Commerce, and Business Service (ACB), 3) Food, Agriculture, and Hospitality (FAH), and 4) Technology, Construction, and Transportation (TCT). The objective of the above-mentioned intervention study was to develop an intervention offering, among other things, alternative social activities to smoking at VET schools. The intervention did not show an effect on the number of daily smokers; however, fewer students who smoked occasionally started smoking daily [18]. To develop more effective interventions and inform policy in terms of smoking reduction and prevention among this high-risk group of VET students, further research is needed to explain the smoking practices, experiences, beliefs, and social norms among low SES youth in the VET school settings and to identify the factors legitimizing smoking at VET schools [20, 21].

Based on participant observations combined with individual and group interviews with students, teachers, and managers from VET schools, the current article aims to explore the smoking practices, experiences, beliefs, and social norms among low SES youth in the VET school setting with a point of departure in the following research question: What are the major factors sustaining legitimacy of smoking in Danish VET schools, and hence the most important to consider when developing and implementing a smoking intervention in this setting?

## Methods

### Sampling

The VET-system consists of a basic program followed by a main program [19]. To gain access to the VET setting, we collaborated with partner-organizations and individuals connected to the VET-system. They functioned as gatekeepers to establish the initial contact with VET teachers and managers. Through the gatekeepers, we established collaboration with three schools representing two of the four main subject areas, namely Care, Health, and Pedagogy (CHP) and Technology, Construction, and Transportation (TCT) (two CHP and two TCT classes). The two areas differed in distribution of sex and age. The one school with two TCT classes were dominated by men, which also affected the sex-distribution within the classes observed. The other two schools each with one CHP class had equal numbers of men and women. By coordinating with the school managers, we were given permission to observe and interview students and teachers in four different classes. In one school we observed a carpentry class and a painting class, while the other two schools each participated with a class studying pedagogy.

### Design

A qualitative study design was used to explore significant factors related to the legitimacy of smoking in the VET school setting. As outlined in Table 1, three methodologies were used, including participant observations at the school setting, individual semi-structured interviews with employees, and focus groups with students.

**Table 1:** Overview of methods and participants

*VET classes*	*Main Subject Area*	*Method*	*Participant Characteristic*
Class 1, School 1 Health Care (CHP)	Pedagogic Assistant	Observation: 5 days	Students and teachers
		Interview: *N* = 3	School manager, teachers
		Focus Group: *N* = 7	Students
Class 2, School 1 Health Care (CHP)	Pedagogic Assistant	Observation: 4 days	Students and teachers
		Interview: *N* = 1	Student Advisor
		Focus Group: *N* = 7	Students
Class 3, School 2 Technical (TCT)	Carpenter	Observation: 4 days	Students and teachers
		Interview: *N* = 1	Teacher
		Focus Group: *N* = 4	Students
Class 4, School 3 Technical (TCT)	Painter	Observation: 3 days	Students and teachers
		Interview: *N* = 1	Teacher
		Focus Group: *N* = 2	Students

*Participant observations* were used to explore the everyday social interactions between the students as well as between students and teachers. Observations were conducted to study tacit knowledge and circumstances in the context which may be taken for granted by participants [22, 23].

*Focus groups* were chosen to explore participants’ opinions and perspectives on smoking. The objective was to launch a dynamic discussion between the participants to explore their negotiations of the meaning and significance of smoking in the VET setting, and to observe their interactions and mutual responses to the unfolding statements, experiences, and opinions [24].

*Individual semi-structured interviews* were suitable to explore and gain insight into individual experiences and thoughts about specific topics and questions of interest, preconceived or appearing from the observation data [25, 26]. We used individual interviews to explore how teachers and managers experienced smoking at the VET schools in general, as well as their perception of their own (lack of) smoking. In addition, we focused on their reflections about potential future smoking prevention and cessation interventions within a VET school setting to assess the school’s interest and possible engagement in future health promotion interventions. The three methods thus provided supplementary insights to help answer the research question.

### Procedure

The qualitative data was generated between March and June 2017 with similar approach for each of the participating classes starting with participant observation followed by individual interviews and focus groups. In each of the four classes, students and teachers were observed by one researcher (JLV) for three to five days during school hours. In total, 100 students aged 16–34 years were involved in the observations.

Prior to observation, the research team outlined a strategy for observation with a particular focus on the atmosphere, relationships, and social dynamics as well as the organization of school activities, and the physical environment. To guide the researcher during observation, a simple memo sheet was outlined by the research group. The participating observer was passive during class hours, whilst informally engaging and interacted with students and teachers during breaks and before and after school hours. Field notes as sentences, words and drawings were taken during observations and structured with additional reflections later the same day [27]. Post observation, transcribed field notes were discussed with the research team to start forming the analysis and the interview guides for both individual interviews and focus group interviews.

On the last day of observation within each class, both students and teachers were invited to participate in interviews. Interviews were conducted at the school premises during school hours and audio-taped following written consent by students and staff, respectively. Afterwards, a transcript was produced by a student assistant. The duration of the focus groups was 1.5 h, while the semi-structured interviews lasted 30 min to two hours. The focus groups' composition was intended to include six to eight students representing both smokers and non-smokers, but cancellations due to illness and school obligations reduced the number of students in each focus group. School managers and teachers were interviewed individually according to participants’ own preferences. A semi-structured interview guide was developed prior to the interviews, including multiple themes directly or indirectly related to smoking. The themes derived from insights from the participant observation, discussions in the research team based on prior experience, and literature from the field, including evidence from previous similar research studies [28–31]. Despite variation in themes between focus groups (see Table 2) and individual (see Table 3) interviews, we aimed for openness and flexibility to discuss topics and questions relevant to the participants.

**Table 2:** Themes in the semi-structured interview guide with students

*Experiences with and attitudes towards smoking and quitting*
*Well-being at school*
*Expectations for school*
*Smoking regulation and prohibition*

**Table 3:** Themes in the semi-structured interview guide with managers and teachers

*Existing smoking prevention and cessation interventions at the school and conceived relevance of those*
*School influence on developing the interventions*
*Decision making and internal communication on school engagement in public health interventions*
*Implementation of the interventions incl. measures and preparations to make the organization ready for the interventions*

### Data analysis

The analysis was inspired by Malterud’s systematic text condensation [32], which offers a pragmatic approach that enables triangulating transcripts of different types of qualitative data, including interview data and observational field notes. During the fieldwork, the research group discussed ongoing observations and upcoming themes from the interviews. Based on these discussions, JV coded the material. The research group held several analysis workshops to provide input from the other researchers as well as discuss and revise the codes and the synthesized themes. Specifically, we applied codes encapsulating meaning, descriptions, and concepts, both concerning the daily life at VET schools, and focusing more specifically on elements such as smoking norms, visibility of smoking, and the social role of cigarettes. The following process consisted of contrasting and comparing across the themes. Finally, this enabled us to identify some important factors that functioned to sustain legitimacy of smoking practices in the VET school setting as reported in the following result section.

### Ethics

This study complies with the current Danish rules of ethics and legislature of the Danish Data Protection Agency (Ref: 17/12006). Ethical approval was not required, as per National Committee on Health Research Ethics (Ref: 20182000–83). However, to comply with ethically sound research practice, prior to the interviews, participants were informed about the purpose of the study and their right to withdraw at any point in time during or after the research process until the research had been published. Participants received contact information should they have questions or desire that their data be withdrawn. Informed consent was obtained from all participants before initiation of the interviews. Data was pseudonymized and kept in a password-protected computer.

## Results

The analytical themes presented below represent what this study found was four major factors sustaining legitimacy of smoking in Danish VET schools. The themes include 1) the smoking norms at home and among friends, 2) VET professions and visibility of smoking, 3) the social and pedagogical role of cigarettes, and 4) boredom, habit, and stress relief.

### The smoking norms at home and among friends

Smoking practices in the home environment was described as significant for the VET students’ smoking norms – and behavior, and the likelihood and possibility to take up, reduce, or quit smoking. Many students had been exposed to smoking during their upbringing, and smoking was generally described by students as a legitimate and normal practice. In some cases, students were taught to smoke by their parents and parents supported smoking financially, while other students experienced lack of support from their parents to either reduce or quit smoking. As one student explains:

*“He [stepdad] keeps asking me, if for instance we go grocery shopping, if I want one [a cigarette]. If I say no, he asks ‘are you sure?’. Then I say, ‘yes I’m sure’ but then he hands it to me anyway”.*

*(Student focus group)*

Indirect pressure from friends also influenced some students’ smoking initiation, and their ability and motivation to start or refrain from reducing or quitting smoking. For instance, acceptability of smoking dominated peer relationships as exemplified by the following quote, where a student described his process of becoming a daily smoker:

*“I started smoking early, I guess I was twelve or thirteen… But it was my buddies who were older than me, right, they smoked, and then I also started smoking. So, I think I started smoking on a regular basis at fifteen or something like that…” (Student focus group).*

In general, smoking represented an acceptable and normal social practice among the students with smoking being described as an integral part of many students’ everyday life at home and among friends.

### VET professions and visibility of smoking

Our observations and interviews revealed that the students generally associated VET schools and the related VET professions with smoking. This contributed to a general perception among the students that smoking represented both an accepted and expected behavior in this setting. Consequently, smoking legislation or stricter rules were not considered realistic, appropriate, or desirable by those studying or working at the observed schools.

#### VET schools and professions were associated with smoking

Students often associated VET schools and professions with smoking. These social beliefs especially applied to technical professions, but also to health care and pedagogical professions. One student in a focus group explained:

*“I think there’s a lot who take up smoking [during VET training] because craftsmen, they really smoke.”*

*(Student focus group)*

The quote illustrates how students expected smoking to be a widespread practice in VET schools but also in the occupations of which they studied to become a part. Another student who also associated VET professions with smoking explained:

*A lot of craftsmen smoke, I think 70%. They all walk around with a cig in their mouth!” (Student focus group).* It was a common perception among students that the professional identity of many VET professions was associated with smoking. This association was further strengthened by their own experience at the school, as two students discussed:

*“I think 80% [at the school] smoke” and “Almost everybody [at the school] smokes”. (Student focus group)*

These perceptions of high smoking rates underscored the strong presence and legitimacy of smoking and illustrated how students experienced smoking as an integral part of the VET school environment, including their future professional identify.

#### Visibility of smoking

Smoking is often visible upon arrival at Danish VET schools. Our observations revealed that students who smoked gathered in specific physical places near the school. This was particularly the case in schools with a ‘smoke-free premises’ policy in which smoking was permitted outside official school premises only. At schools with this policy, students gathered in smaller, demarcated areas, often just a few meters away from the main entrance of the school. Consequently, greater visibility of smoking was instigated, and the demarcated area in front of the school became associated with smoking as a dedicated and legitimate ‘smoking area’. As two students put it:

*“It’s a bad idea to place all the smokers in the same spot!” (Student focus group)*

*“It’s like, as soon as you go out there you want a smoke, because that place is for smoking – that is, if you are out there, you are there to smoke, that’s how it is, because that’s where the smokers are.”*

*(Student focus group)*

As smoking was visibly practiced in the school environment, students tended not to question or reflect on their smoking practices:

*“You do what others do (…) and you don’t do what others don’t do. We are pack animals!” (Student focus group).*

In this respect, the visibility of smoking appeared to enhance the existing norms and expectations related to smoking in the VET school environment. Moreover, the ‘smoke-free premises’ policy rather seemed to increase visibility of smoking and strengthen the perception of smoking as legitimate and acceptable.

### The social and pedagogical role of cigarettes

Cigarettes were also an essential element in making and maintaining social relations at VET schools, whether it was between friends or when connecting with new people, such as other students or teachers.

#### Smoking facilitates social relationships

Most new students enrolled in a youth educational program expect to make new friends and engage in new meaningful social relations – on personal and professional/academic levels. In the process of making new friends, cigarettes can play a significant role. The social role of cigarettes was highlighted by several students explaining how smoking was used to establish social relationships, both between peers and with teachers:

*“If you smoke and you see others who smoke, then you already have something in common” (Student focus group)*

Smoking functioned as a point of reference by creating a shared sense of community and identity among the students. For the younger students, in the transition phase from elementary school to VET, this appeared to be particularly important. A student described the awkwardness associated with making friends when starting at a new school:

*“It just seems odd, weird, to go over and say’hi’. How will you go on with the conversation from there?” (Student focus group)*

To deal with this awkwardness, cigarettes were described as an easy and legitimate ‘tool’ to break the ice and make new friends:

*“If you just started in the same class and you get a smoking break, then you just join the others and pretend you don’t have a lighter, then you are already talking to someone.” (Student focus group)*

Smoking was described as a helpful way to approach new classmates, and cigarettes provided a conversation topic and a sense of belonging in an otherwise awkward situation. The need to build relations in a new school thus enhanced the need to smoke.

#### Teachers are role models

Teachers who smoked also played a significant role in legitimizing smoking practices. Teachers are role models for students aiming to acquire a professional identity associated with their profession. As such, teachers represented the example to follow, also when they did not comply with the school’s smoking policy themselves. One example was some teachers who circumvented existing smoking regulations by smoking in the demarcated area. As a student explained:

*“I find it hard to comply [with the smoking rules] when those who say you can’t smoke in front of the school do it themselves. Then you don’t want to comply with the rules either”. (Student focus group)*

During breaks, it was a widespread practice for teachers to smoke alongside the students. For instance, one teacher was observed rolling cigarettes during class in front of the students. And, on several occasions, teachers were observed referring to breaks as “*cigarette breaks*”. Teachers thus reinforced smoking as a normalized and integrated everyday practice:

*“Well, the teachers are kind of encouraging smoking when they say:’well, let’s take a cigarette’ or’now, you can go for a cig and come back in five minutes’. You know, it’s kind of in your head that now you need to go and have a cigarette.” (Student focus group)*

A student even explained how their teacher had instructed the students to circumnavigate the rules:

*“He [the teacher] told us’Well, don’t tell them that you smoke here. Just do it without me knowing about it’”. (Student focus group)*

Particularly teachers who smoked themselves did not enforce smoking regulations and, in some instances, even encouraged smoking. Teachers’ own smoking practices and active articulation of smoking as an institutionalized legitimate practice thus functioned to legitimate students’ smoking.

#### Fear of jeopardizing pedagogical relations

From the teachers’ perspective, another important factor that was expressed in the interviews was the need to build and maintain a trustful and equal relationship with students. This relationship was of particular concern to the teachers because VET students often came from low socioeconomic backgrounds, and many students struggled with psychosocial issues and low motivation for schoolwork [11], which made teaching in this setting pedagogically demanding. From the teachers’ perspective, building a trustful relationship was thus fundamental. Many teachers worried that a role as “*smoking police*”, which enforcement of smoking regulation might imply, would jeopardize their good pedagogical relations and the mutual trust needed for teaching the students. Accordingly, teachers feared that sanctioning students’ smoking could threaten the positive teacher-student relationships as expressed by one teacher:

*“Building a personal relationship with the student is extremely important. And I don’t want to jeopardize this relation by acting ‘police’.” (Teacher interview)*

In this respect, the responsibility, and obligations to abide by smoking regulations collided with other more important obligations, as another teacher explained:

*“Our relationship with the students will suffer if we confront them all the time”*. *(Teacher interview)*

In addition, teachers who were smokers themselves experienced certain advantages related to smoking alongside the students since the informality in these social situations represented good opportunities to build better relationships. One teacher expressed how the mutual experience of craving a cigarette provided a common ground between students and teachers who smoked:

*“We have something in common, we do, we recognize the craving, you know, that we simply need a cig every once in a while”. (Teacher interview)*

The same teacher went on to express and describe a mental distance between smokers (‘us’) and non-smokers (‘them’), at least in the view of non-smokers:

*“… for someone like Jens [another teacher who did not smoke], this is impossible to understand”*. *(Teacher interview)*

To sum up, smoking was understood as an easy way to make new friends. From the students’ perspective, teachers were significant role models, and the attitude of the teachers towards smoking seemed significant to whether students chose to comply with school tobacco regulation. In this respect, teachers choosing not to sanction smoking, to smoke themselves, or to encourage smoking in the company of students may reinforce the legitimacy of smoking at the school.

### Boredom, habit, and stress relief

A final factor legitimizing smoking was associated with the motivation for schoolwork among students; the potential stress schoolwork sometimes cause for some students; the lack of opportunity for students to engage in non-smoking social activities; and the addictive properties of smoking.

#### Boredom and habit

At the VET schools investigated in this study, the possibilities for students to engage in social or physical activities during breaks were limited. Instead, students described how smoking was used to pass time, as a diversion to avoid boredom due to lack of other potential activities:

*“Well, there are two options: You can stay inside in the canteen and talk, or you can go outside to smoke”*. *(Student focus group)*

In addition, some students explained how smoking had become a habit and part of their daily routines:

*“It is like when people need coffee – it’s [smoking] just a habit”*. *(Student focus group)*

Other students expressed how cigarette breaks during school hours were often due to habit rather than an active choice, underpinning a lack of alternative offers. In relation to habit, some students claimed that they were able to quit smoking if they wanted to; a key argument for not doing so was a question of lack of motivation. Others gave an account that could be interpreted as nicotine dependence:

*“You get a nice feeling in the body”* and “*they [the cigarettes] make you relax*”. *(Student focus group)*

#### Soothes stress and negative feelings

Nicotine dependence appeared to be related to how several students also explained their use of cigarettes to soothe stress in various situations:

*“They calm you down”, “I feel at peace when I smoke”.*

For instance, some students used the cigarettes to initiate a short (mental) break when things went awry in class:

*“If there is a conflict in here and you, for instance, just need to go outside for a smoke, it helps, it makes you calm down and relax – and then you can go back inside and be calm.” (Student focus group)*

Also, several students explained how cigarettes helped them in stressful situations or as a relief from mental distress, as one student expressed:

*“If I don’t feel well, my cig will help. Nothing else works”. (Student focus group)*

Hence, in addition to boredom and habits, the students associated smoking with stress relief, using cigarettes to deal with both stressful situations and difficult emotions. This underpinned smoking as a rooted habit or outright dependence among students who smoked. In addition, the use of cigarettes to suppress boredom and anxious or depressive feelings further added to sustain legitimacy of smoking among VET students.

## Discussion

The aim of this qualitative needs assessment was to explore the smoking practices, experiences, beliefs, and social norms among low SES youth in Danish VET school settings to identify the major factors sustaining legitimacy of smoking. Our findings reveal that participants in this study had positive attitudes towards smoking reflecting that neither at home nor in school was smoking taboo or an illegitimate practice. This highlights the influence of norms and health behavior of significant others, such as parental smoking and smoking rules at home, on the initiation of smoking [33, 34]. Importantly, our study showed the pervasive normalization of smoking in VET schools, where it is intertwined with students’ present and future professional identity. The visibility of smoking at the school premises contributes to a “smoking narrative” in VET schools [35]. Hence, a smoke-free school ground policy may counteract the intent to reduce smoking and rather encourage or even initiate smoking [36, 37]. The visibility also led to a misconception of high smoking prevalence among students. This “pluralist ignorance” may further contribute to smoking being perceived as a natural part of everyday life in a VET school and can play a role in how students accept and apply social labels, which further reinforce smoking [38, 39].

Younger students emphasized that smoking was an ice breaker, and an easy means to contact others, to make new friends when initiating the educational program. Young people’s need to feel like they belong at school may entail that they use smoking as a social strategy to ameliorate the risk of alienation [40]. A systematic review [41] examining qualitative research on smoking among disadvantaged young people found that smoking represented a marker of identity entrenched in social inclusion processes and thus a “currency of social capital” (41:432). Parallel to this process of socialization into smoking was the parental acceptance of smoking and hence a lack of authorities in the young people’s lives to question the social acceptance of smoking [41]. Similarly, in this study, the teachers emphasized the advantages of smoking during breaks and in other situations to create good relationships with students, who were otherwise experienced as difficult to reach, and they were reluctant to jeopardize their relationship with the students to uphold smoking regulations. As important role models, the teachers thus played a contributing role in legitimizing smoking. This supports previous findings which underscore that role models influence health-behaviors in youth, including smoking [42, 43]. The experiences of the participants in our study suggest that school staff have a prominent role in creating smoke-free school environments. The staff at youth education institutions have been found to value a positive teacher-student relationship highly [28, 44]. A positive teacher-student relationship is the foundation for teachers’ abilities to teach, and sanctioning students smoking is therefore often assumed to threaten this important relationship [28]. Moreover, many teachers and other school staff are smokers themselves and may not support potential regulation introduced to reduce smoking.

Other studies have found that the legitimacy of smoking exists due to students’ lack of motivation for schoolwork, the lack of possibility to engage in other (social) activities than smoking, as well as the habitual and addictive properties of regular smoking. As has also been found in previous studies, smoking has perceived benefits including getting breaks and dealing with stress and boredom [29]. This could lead to increased consumption and counteract interventions to reduce smoking prevalence and may even encourage non-smokers to initiate smoking.

### Implications for practice

The identified factors legitimizing smoking are consistent with previous research on youth smoking, though our findings add new perspectives to the current practices in a Danish VET school setting. The identified factors reciprocally enforce each other in how they reproduce smoking norms, perceptions, and attitudes to smoking. Hence, a major challenge, when planning and implementing smoking prevention and reductions, is the interplay between the factors and the reciprocity created in the social processes of everyday life in VET schools.

In this study, we identified a specific need to influence the social norms as well as the social and physical environment in the VET school setting. For instance, teachers’ smoking practices and attitudes, the visibility of current smoking practices and the lack of rule -or legislation enforcement. In addition, we found that alternative social activities during breaks are needed to encourage social relationships which are not centered around or determined by smoking. Finally, there is a need to address the habitual and soothing role of cigarettes. In this respect, psychosocial support is necessary to assist students who use cigarettes during school-hours to cope with challenges due to school-related or personal problems, or dependence.

Accordingly, our findings suggest four key measures to prepare implementation of smoking prevention and reduction interventions in Danish VET schools. First, and most importantly, the intervention should change school policies by introducing a stricter tobacco regulation than smoke-free premises, e.g., a smoke-free school hour policy [45]. If properly implemented, this regulation is likely to reduce visibility of smoking, and, over time, may contribute to changing the existing perceptions of smoking as a normal and expected practice in VET schools. Secondly, measures are needed to persuade teachers that smoking prevention and smoking regulation are needed and important to their work, emphasizing that productive pedagogical relations with students can exist without the use of smoking breaks and socializing using cigarettes. This implies attention to the smoking practices of the teachers and necessitates that both school managers and teachers accept and prioritize enforcement of smoking regulations during their working hours. Thirdly, providing students with alternative views and perceptions of smoking and altering the possible overestimation of smoking practices at school is important. This overestimation is known to affect the likelihood and desire to initiate or consider smoking. Alternative social activities should be available to offer students in VET schools the opportunity not to smoke a better one. Finally, measures should include help and support (i.e., nicotine substitutes or counseling) for students as well as employees to help them reduce or quit smoking, or abstain from smoking during school days, to counteract the positive functions associated with stress relief and nicotine dependence.

The above measures are comprehensive and resource-demanding and thus, none of the above are easily introduced or implemented. This is further challenged by lack of prior experience with smoking regulation within the VET school setting, and not least limited resources within the schools. Still, our study shows that there is no simple solution to prevention or reduction of smoking in youth populations. Accordingly, changing existing smoking norms is realistic only within a long timeframe, and if relevant actors and stakeholders around the VET school and students are motivated for change. But initiating a change in attitudes towards smoking and regulations on smoking is an important first step.

Lastly, some considerations must be made regarding potential negative consequences of tobacco regulations and norm changes. For instance, low SES students are at risk of experiencing marginalization, thus reinforcing inequality and affecting the most exposed or smoking-dependent students. Also, tobacco regulation may induce negative consequences for the student–teacher relationship, which may further marginalize low SES students already at risk of not completing an educational program. Finally, implementation of a stricter tobacco regulation policy implies extra pressure and use of resources among VET teaching staff who already experience conflicting demands and time scarcity. In this respect, continuous ethical considerations are important at each step of the process while developing a smoking prevention/reduction intervention.

### Strength and limitations

A key strength of this study is its contribution to elucidate and nuance an important field in smoking prevention literature, including low SES youth and the VET school setting, which is limited and inadequately investigated despite high smoking prevalence. Also, our data material contains observations at multiple VET school settings, and includes the perspectives of both students, teachers, and managers through interviews enabling comprehensive insight to the field of study. The purpose of this article was to identify the major factors sustaining legitimacy of smoking; however, we acknowledge the relevance of also exploring potential drivers for smoking prevention and reduction interventions and the social, cultural, and political forces affecting these, which were delimited from this paper. Also, we did not focus on snuff or e-cigarettes, which are closely related to the use of cigarettes, and have been highlighted as a major health problem among youth more recently [46]. Moreover, our findings are derived from few VET schools representing only half of the four main subject areas in the Danish VET-system. As we discovered, the physical environments of VET institutions are diverse, and the psycho-social characteristics of the students differ across and even within the four main subject areas. Lastly, the Danish VET school setting is continuously undergoing structural transformation and reform. Thus, reservations must be made in relation to the generalization to other main subject areas, student populations, organizational structure, etc. This reservation also applies to VET schools in other countries. Finally, it is important to address that this study was conducted before the onset of the national legislation ‘Smoke-free school hours’ (SFSH), which was implemented by law in Danish youth education institutions by July 31st, 2021. According to the law, smoke-free school hours include all tobacco-related products, and it regulates students’ behavior, while school staff is still allowed to use the products outside school grounds during school hours. At the time of this study, the legislation was neither planned nor activated, hence it did not apply to VET schools where tobacco regulation relied on the individual school. Despite the subsequent smoke-free school hours legislation, recent Danish data indicate that a significant majority of students who use tobacco products still smoke cigarettes (88–92%) or use smokeless tobacco (96–98%) during school hours [46]. Similar findings are reported in other European countries [47]. In addition, a recent Danish report evaluating implementation of SFSH at different youth education sites in The Region of Southern Denmark [48] underscores the challenges attributed to resource constraints and other factors likewise mentioned in this paper. Future studies might use the findings from the current paper to explore new research questions and implementation studies, which can be used to assess the challenges and facilitators in implementing SFSH and contribute to the ongoing efforts in tobacco control. Moreover, future studies can explore alternative interventions to complement legislative measures, which may include educational programs or support services.

## Conclusions

This qualitative need assessment elucidated complex and varied experiences of smoking among students in the Danish VET school setting. Collectively, the identified factors point to the need for a strict tobacco regulation that incorporates the smoking practices of teachers and other school staff, as well as a focus on the social environment in VET schools. Interventions should be designed to acknowledge and address students’ psycho-social and academic needs and include a focused effort to enhance the capabilities of those who experience nicotine dependence or psychological addiction.

## Data Availability

The dataset used and analysed during the current study is available from the corresponding author on reasonable request.
